# RON (*MST1R*) and HGFL (*MST1*) Co-Overexpression Supports Breast Tumorigenesis through Autocrine and Paracrine Cellular Crosstalk

**DOI:** 10.3390/cancers14102493

**Published:** 2022-05-19

**Authors:** Brian G. Hunt, Angelle Jones, Carissa Lester, James C. Davis, Nancy M. Benight, Susan E. Waltz

**Affiliations:** 1 Department of Cancer Biology, University of Cincinnati College of Medicine, Cincinnati, OH 45267, USA; huntbg@mail.uc.edu (B.G.H.); jones8ad@mail.uc.edu (A.J.); lestercn@ucmail.uc.edu (C.L.); davis8je@mail.uc.edu (J.C.D.); benight12@gmail.com (N.M.B.); 2 Research Service, Cincinnati Veterans Affairs Medical Center, Cincinnati, OH 45220, USA

**Keywords:** receptor tyrosine kinases, macrophage activation, tumor microenvironment, breast tumorigenesis

## Abstract

**Simple Summary:**

RON is a protein that sits on the surface of a cell and passes signals from the outside-in when encountering the HGFL protein. In cancer, the RON protein is often found at high levels leading to too many signals being passed. Given the close proximity of RON and HGFL in the human genome, we hypothesized that the two proteins are overproduced together in cancer cells. Other cells that interact with cancer cells, such as macrophages, also express RON and can respond to HGFL produced by cancer cells, creating a diverse response to overly abundant RON and HGFL. In this study, we evaluated how RON and HGFL in cancer cells affect cancer growth and progression and their response to other cells, and examined explanations for increases in RON and HGFL abundance in breast cancer.

**Abstract:**

Background: Aberrant RON signaling is present in numerous cancers including breast cancer. Evidence suggests that the ligand, hepatocyte growth factor-like (HGFL), is also overexpressed in breast cancer. RON (*MST1R*) and HGFL (*MST1*) genes are located on human chromosome 3 and mouse chromosome 9 respectively and are found near each other in both species. Based on co-expression patterns, we posited that RON and HGFL are co-regulated and that coordinate upregulation drives aggressive tumorigenesis. Methods: Mouse models were used to establish the functional significance of RON and HGFL co-overexpression on the activation of tumor cells and tumor-associated macrophages in breast cancer. TCGA and METABRIC gene expression and alteration data were used to query the relationships between *MST1R* and *MST1* in breast cancer. Results: In tumor models, physiologic sources of HGFL modestly improve Arginase-1^+^ (M2) macrophage recruitment to the tumor proper. Tumor-cell produced HGFL functions in autocrine to sustain tumor cell RON activation and MAPK-dependent secretion of chemotactic factors and in paracrine to activate RON on macrophages and to promote breast cancer stem cell self-renewal. In silico analyses support that RON and HGFL are co-expressed across virtually all cancer types including breast cancer and that common genomic alterations do not appear to be drivers of RON/HGFL co-overexpression. Conclusions: Co-overexpression of RON and HGFL in breast cancer cells (augmented by physiologic sources of HGFL) promotes tumorigenesis through autocrine-mediated RON activation/RON-dependent secretome changes and paracrine activation of macrophage RON to promote breast cancer stem cell self-renewal.

## 1. Introduction

The RON receptor tyrosine kinase is expressed at low levels in epithelial cells and macrophages and is phenotypically responsible for signals to promote wound healing and the resolution of inflammation [1,2,3]. RON upregulation has significant oncogenic implications promoting cell growth, survival, migration, recruitment of pro-tumor immune cells, and suppression of anti-tumor immunity [4,5,6,7,8,9]. Hepatocyte growth factor-like (HGFL) protein, the RON ligand, ligates to two RON homodimers, leading to conformational changes that induce autophosphorylation of the tyrosine kinase (TK) domain and subsequent activation of a plethora of downstream signaling pathways [6,10,11]. RON overexpression is also able to elicit activation through spontaneous receptor dimerization [12] which is sufficient to induce the malignant transformation of mammary epithelial cells leading to metastatic breast tumorigenesis in mice [4]. Targeted deletion of HGFL in mice impairs RON-driven breast tumorigenesis, metastatic progression, and restores anti-tumor CD8 T-cell function, supporting an important role for ligand-dependent RON signaling in oncogenic RON activation even in the context of RON overexpression [5].

RON overexpression occurs in >50% of all human breast tumors [13] and is a predictor of early death, metastatic progression, and recurrence in breast cancer patients [14,15]. Noteworthily, RON overexpression is predictive of outcome across all breast cancer subtypes [14] suggesting the utility of this expression as an independent prognostic indicator for a spectrum of breast cancer patients. In addition to human breast cancers, RON upregulation is also observed in the murine autochthonous Polyoma Middle T-antigen (PyMT) induced mammary tumor model [16]. Strikingly, whole-body loss, mammary epithelial-specific loss, or macrophage-specific loss of the RON TK signaling domain ablate mammary tumorigenesis and metastatic progression in the PyMT model [7,16,17]. These data show that RON signaling in both mammary epithelial cells and macrophages serve as major drivers of breast cancer.

Under normal physiological conditions, HGFL is primarily expressed by hepatocytes and is secreted as a pro-hormone into the bloodstream where it acts in an endocrine fashion to activate RON signaling [18]. A growing body of evidence suggests that HGFL, in addition to RON, becomes overexpressed in tumor cells [5,6,19]. This raises questions as to the functional contribution of the different sources of HGFL, namely physiologic HGFL (primarily secreted from hepatocytes) and tumor cell-produced HGFL, on RON activation. Noteworthily, tumor cell-produced HGFL may function in an autocrine fashion to activate RON on the tumor cell itself or may act in a paracrine fashion to activate RON on macrophages in the tumor microenvironment (TME) during breast tumorigenesis. Interestingly, the RON gene (*MST1R*) and HGFL gene (*MST1*) are cytogenetic neighbors located on chromosome 3p in humans (just over 200 kilobases apart) and chromosome 9q in mice (<200 kilobases apart). Given this association and the increased expression in cancer, we posited that *MST1R* and *MST1* genes may be co-regulated and that certain genomic alterations may dually affect expression. Mechanisms underlying RON and HGFL overexpression in cancer are unknown and recurrent genomic alterations or mutations associated with either or both genes have not been firmly established.

The well-characterized murine PyMT breast cancer model develops multifocal mammary tumors that are luminally biased but highly metastatic [20,21]. Using this PyMT model, we previously showed reduced mammary tumor growth and metastasis in mice with a conditional loss of RON in either mammary epithelial cells or macrophages [7,17]. We also showed that RON expression in either cell type was associated with the presence of pro-tumorigenic macrophages in the TME [7,17]. Tumor-associated macrophages (TAMs) exist on a spectrum of functional plasticity ranging from M1 (pro-inflammatory, anti-tumor) to M2 (anti-inflammatory, pro-tumor) which is greatly influenced by various cues in the TME [22,23]. Herein, we sought to understand the contributions of the different sources of HGFL on mammary tumorigenesis. Our studies show that HGFL expression, regardless of the source, supports mammary tumorigenesis. Further, HGFL that is upregulated in mammary tumor cells supports breast cancer phenotypes through actions on RON in both tumor cells and macrophages of the TME. Physiologic (primarily hepatocyte-derived and secreted) HGFL plays a role in honing macrophages to the TME and in preventing the recruitment of adaptive immune cells. Tumor cell-produced HGFL functions in autocrine to activate RON and downstream MAPK signaling in the tumor cell, which leads to the production of cytokines/chemokines that draw Arginase-1^+^ macrophages into the TME. Tumor cell-produced HGFL also acts locally in a paracrine fashion on macrophages to elicit cytokine secretion that dampens anti-tumor immunity. Both autocrine and paracrine mechanisms support mammosphere/breast cancer stem cell formation. We also evaluated the extent and putative mechanisms underlying *MST1R*/*MST1* co-overexpression. Utilizing genomic approaches, we show coordinate overexpression of both proteins in almost all human cancers. Our studies further suggest that RON and HGFL are rarely altered in human cancers and upregulation is not associated with common breast cancer driver mutations. Finally, we demonstrate the anti-cancer utility of RON pharmacological inhibition in targeting breast cancer cells and the TME to limit mammary tumor growth and progression.

## 2. Materials and Methods

### 2.1. Mice

Female mice were used in all experiments and all mice were in an FVB/N background. RON tyrosine kinase (TK) domain wild type (WT/TK^+/+^), RON TK domain deficient (TK^−/−^; MMRRC #067438), HGFL deficient (HGFL^−/−^), Lysozyme M-Cre (Lys-Cre^+^; Jackson Laboratories, Bar Harbor, ME, United States, Strain #004781), MMTV-RON (MMRRC #067439-UNC), and PyMT mice (Jackson Laboratories; Strain #002374) have been previously described [2,6,16,24]. Mouse genotyping primers are as follows: PyMT#1 5′-GGAAGCAAGTACTTCACAAGGG-3′; PyMT#2 5′-GGAAAGTCACTAGGAGCAGGG-3′; HGFL#1 5′-AATCTGGGTTGCCAGTTAACTTTGTG-3′; HGFL#2 5′-AAGTTCTCTTCCAGGCCATTCTTTGG-3′; HGFL#3 5′- GGAAAAGCGCCTCCCCTACCCGG-3′; RON TK^−/−^#1 5′-TCATTTGAATCAGTCCCCTCACTTTTCTCC-3′; RON TK^−/−^#2 5′-GGAACCAGTACACAGATGAGTAAACTGAGC-3′; RON TK^−/−^#3 5′-TCGCTCAAGCCCAGGCAGGGCCTCACAGAG-3′; Lys-Cre#1 5′-TTACAGTCGGCCAGGCTGAC-3′; Lys-Cre#2 5′-CTTGGGCTGCCAGAATTTCTC-3′; Lys-Cre#3 5′-CCCAGAAATGCCAGATTACG-3′; MMTV-RON#1 5′-TGGGTGGTGAGGTCTGCCAACATGAGCTCC-3′; MMTV-RON#2 5′-CCGTCTTCGGGAGTTAAAGATCAGGGCAAC-3′. RON inhibition in WT PyMT mice was performed using 50 mg/kg/day BMS777607 (Selleckchem, Houston, TX, USA) treatment via oral gavage [25], starting at 8 weeks of age (when tumors reached a measurable size). For orthotopic transplantation studies, female mice between 6–8 weeks old were anesthetized, a small incision was made to reveal the mammary gland, and 150,000 cells in 50 µL were injected into the gland [6,7]. Macrophage depletion in WT (HGFL^+/+^) or HGFL^−/−^ mice was performed using 200 µL clodronate encapsulated in liposomes (SKU# CLD-8901 Encapsula Nano Sciences, Brentwood, TN, USA) injected intraperitoneally beginning 1 day prior to tumor cell implantation and continuing with 3–4 doses weekly for the duration of the study [26]. Mammary tumors were measured using calipers and tumor volume was calculated as previously described [27]. Total tumor burden in PyMT mice, which typically develop tumors in all mammary glands, was calculated by adding individual tumor volumes together.

### 2.2. Histology and Immunohistochemistry (IHC)

Harvested tissues including mammary tumors and lungs were processed as previously described [4,5,16]. Hematoxylin and Eosin (H&E) and IHC staining for RON, HGFL, BrdU, Ki67, TUNEL, F4/80, Arginase-1 (Arg-1), iNOS, Cleaved Caspase 3 (CC3), CD4, and CD8a were performed as described [5,7,24]. Scoring was performed on a minimum of 3 independent fields per tumor section from at least three independent tumor samples. The lung metastatic area was determined using a scale bar to calibrate measurements from H&E stained lung sections and by using the freehand selection tool on ImageJ to trace and quantify the metastatic area [28].

### 2.3. Cell Cultures

The R7 murine mammary tumor cell line was derived from a mammary tumor harvested from an MMTV-RON^+^ female mouse [4] and has been extensively characterized [5,6,7]. R7 sgRON cells are R7 cells with RON loss via CRISPR-Cas9 technology (R7 KD cells) as described previously [7]. Bone marrow isolation was performed as previously described [29] with differentiation to bone marrow-derived macrophages (BMDM) via the addition of macrophage colony-stimulating factor (MCSF). MCSF-containing media was produced from CMG1412 cells (obtained from Dr. Yi Zheng) as previously described [30], and was applied to bone marrow for ten days for complete BMDM differentiation. BMDMs were polarized to a tumor-associated macrophage (TAM)-like state by adding recombinant murine IL-4 (Peprotech Cat#214-14) and used for experimentation as previously described [17]. Conditioned media (CM) was produced through the replacement of serum-containing media for serum-free media (SFM) on cells seeded at an equal density (across treatment groups). CM was collected at 24 h (tumor cells) or 48 h (BMDM). Cells were confirmed negative for mycoplasma through PCR before experiments.

### 2.4. Mammosphere Formation Assays

Mammosphere formation assays were performed by plating 50,000 R7 cells in non-adherent cultures using serum-free mammosphere media as previously described [6]. Mammospheres were either monocultured or co-cultured at a 1:1 ratio with BMDMs from either wild type (WT, TK^+/+^) or RON TK deficient (TK^−/−^) mice [6]. Recombinant HGFL (rHGFL; Cat# 4306-MS/CF R&D Systems) supplementation was performed by daily addition of 50 ng rHGFL to respective cultures. After 10 days of culture, images of mammosphere-containing wells were captured and mammospheres > 50 microns in diameter were counted using ImageJ software [28]. Sphere size was calculated using a scale bar-calibrated line tool to measure each sphere. The number of spheres greater than the cutoff value was then compared between groups.

### 2.5. Migration Assays

Migration assays of WT BMDMs toward SFM or CM from R7 cells with or without shRNA targeting of HGFL (1:1 dilution ratio of BMDM CM to SFM), or co-culture with R7 or R7 shHGFL cells (1:10 ratio of R7 cells to WT BMDM cells) using 4000 R7 cells and 40,000 BMDM cells were performed for up to 6 h using Boyden chambers [5]. For studies involving inhibitors, R7 cells were pretreated with inhibitors including 20 µM Ravoxertinib (ERK1/2 inhibitor; MedChemExpress, Monmouth Junction, NJ, USA, Cat#50-187-3397), 20 µM Afuresertib (Akt inhibitor; MedChemExpress Cat#50-187-3338), 20 µM STAT3 Inhibitor VII, S3I-201 (Calbiochem Cat#573103), or 10 µM Bay 11-7085 (EnzoLifeSciences, Farmingdale, NY, USA, Cat#573103) for 6 h prior to washing out and addition of fresh media, and addition of BMDM-containing Boyden chambers. The number of BMDMs that migrated toward SFM was used as a reference control. Following incubation, chambers containing migrated cells were removed, and the cells were fixed and stained using 0.5% *w*/*v* Crystal Violet (Sigma, Sofia, Bulgaria, Cat#C6158) in methanol. The upper part of the chamber was gently wiped clean using a cotton tip applicator to remove non-migrated cells prior to imaging and counting transmigrated cells.

### 2.6. Immunoblot Analyses and Cytokine Array

Lysates for western analyses were obtained by standard RIPA buffer-mediated lysis with the use of cell scrapers to facilitate the complete removal of the cells from the culture plate. RIPA buffer contained protease inhibitors (cOmplete Protease Inhibitor Cocktail; Roche #11 836 153 001; Sigma, Sofia, Bulgaria) and phosphatase inhibitors (PhosSTOP; Roche #04 906 837 001; Sigma, Sofia, Bulgaria). Antibodies for western analyses included: RON (C-20 clone; Cat#SC322, SCBT), HGFL (Cat#SC6088, SCBT), and C4-ACTIN (Cincinnati Children’s Hospital Medical Center, Cincinnati, OH, USA). Secondary antibodies were developed as described [6]. CM from R7 cells or BMDMs was assessed as per the manufacturer’s instructions using the Mouse Cytokine Array XL kit (R&D Systems, Minneapolis, MN, USA, Cat#ARY028). Densitometric analyses of the resulting signals were performed using VisionWorks 7 software (AnalitykJena, Thuringia, Germany).

### 2.7. Bioinformatics

The Cancer Genome Atlas Pan-Cancer (TCGA PanCan) [31] and METABRIC [32] human breast cancer datasets were accessed via cBioPortal (accessed on 12 July 2021) [33,34]. The tumor immune microenvironment estimation resource (TIMER) [35] was used to query relationships between HGFL and RON gene expression with macrophage and T cell infiltration gene signatures.

### 2.8. Statistics

Statistical significance was determined by performing a Student’s *t*-test for two-group comparisons or an ANOVA for comparison of multiple groups; data are visualized as the mean ± standard deviation (SD). Significance (*) was set at *p* < 0.05. Metastasis incidence was analyzed using Fisher’s Exact test. Survival and other time-to-event curves were compared using Log-Rank statistics. Statistical tests were selected based on population distribution, data scale, and sample centrality/variability to meet the assumptions of the tests. The variance between sample groups was found to be similar as tested by ANOVA. TCGA Pan-Cancer data were log_2_ transformed prior to analysis via multiple *t*-tests with the FDR procedure whereas METABRIC data were already log-transformed. Tumor growth kinetics curves were analyzed using a two-way ANOVA with post hoc tests for each time point using the Holm-Šídák method.

## 3. Results

### 3.1. RON and HGFL Overexpression Co-Occurs in PyMT Tumors and Global HGFL Deletion Impairs Tumorigenesis, Metastatic Progression, and Improves T Cell Recruitment

**Figure 1:**
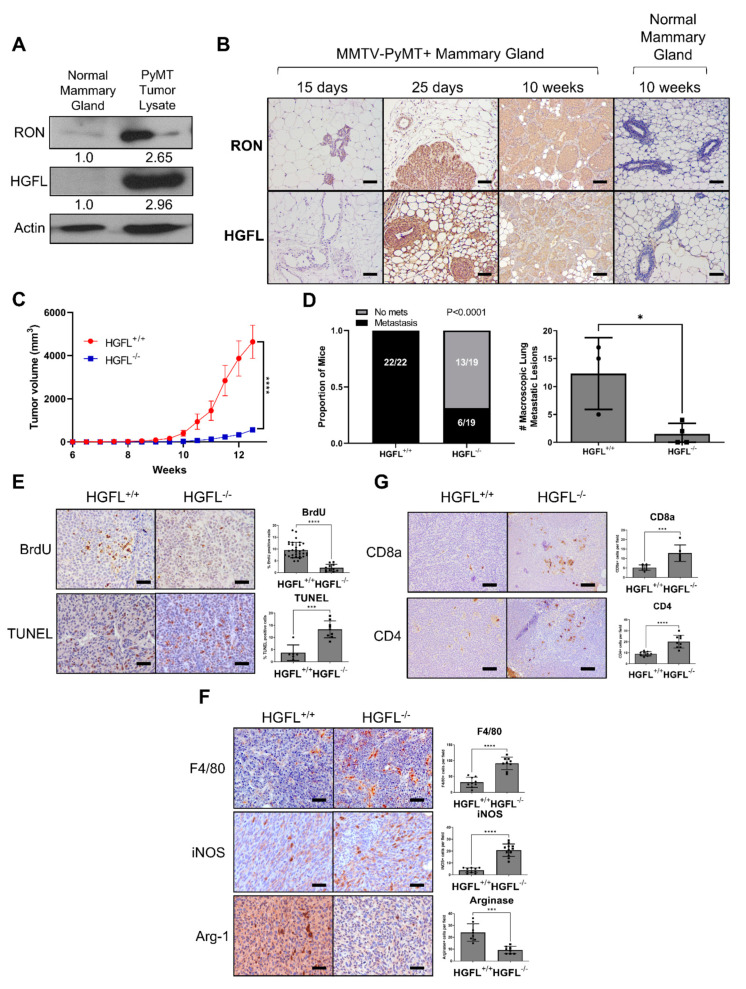
RON and HGFL overexpression co-occur in the murine MMTV-PyMT model of breast cancer and global HGFL deletion impairs tumor growth and metastasis and improves immune cell recruitment. (**A**) Representative western blot for RON and HGFL on lysates from normal mammary glands or PyMT tumor-bearing mammary glands of FVB mice (n = 2). (**B**) Immunohistochemistry staining for RON or HGFL on PyMT tumor-bearing mammary glands or mammary glands of non-transgenic (normal) FVB mice at specified ages (n = 4). (**C**) Tumor growth kinetics of HGFL proficient (+/+; n = 12) or HGFL deficient (−/−; n = 10) PyMT mice and (**D**) respective metastatic incidence and number of metastases in the lungs taken at 90 days of age. (**E**) Representative images and quantitation of specified staining for BrdU and TUNEL, (**F**) F4/80, iNOS, and Arginase-1, and (**G**) CD8a and CD4 in the mammary glands of PyMT (HGFL^+/+^ or HGFL^−/−^) mice at 90 days. Comparisons of histological samples between groups were analyzed using Student’s *t*-tests. * *p* < 0.05; *** *p* < 0.001; **** *p* < 0.0001.

We began with an investigation of the requirement of HGFL in the PyMT mouse model of breast cancer. We previously employed this mammary tumor model to demonstrate cell-type (tumor cell and macrophage) specific contributions of RON [7,16,17]. We also observed RON upregulation in this model and showed coordinate HGFL upregulation in tumor lysates and by immunohistochemical staining from mice as early as 25 days old (Figure 1A,B). Full western blot images are found in Figure S1. Of note, RON and HGFL overexpression is maintained throughout mammary tumor development in virgin PyMT^+^ female mice; normal mammary gland tissue (from age-matched non-transgenic virgin mice) was included for comparison. Similar to the coordinate upregulation of HGFL and RON in human cancer samples, PyMT provides a comparable murine model to examine the functional requirement of RON/HGFL co-overexpression in driving mammary tumorigenesis. PyMT^+^ mice were crossed onto an HGFL^−/−^ background to obtain PyMT mice lacking HGFL (PyMT^+^ HGFL^−/−^). In Figure 1C, we show a significant reduction in mammary tumor growth over time with HGFL loss, and, in examining size matched tumors, we found a significant reduction in incidence and number of lung metastases (Figure 1D). PyMT tumors from mice with global loss of HGFL have impaired cell proliferation (BrdU^+^ staining) and increased cell death (TUNEL staining; Figure 1E). Similar to RON loss in this model [16], mammary tumors from PyMT mice with a loss of HGFL show increases in the recruitment of F4/80^+^ macrophages with increased M1 macrophages marked by iNOS^+^ staining and a reduction in M2 macrophages marked by Arginase-1^+^ staining (Figure 1F). HGFL loss also leads to an increase in T cell recruitment in the TME with more CD8a^+^ T cells and CD4^+^ T cells compared to HGFL^+/+^ PyMT tumors (Figure 1G). Together, these data show that HGFL loss impairs mammary tumorigenesis and alters the TME similar to RON loss in the PyMT model.

### 3.2. Conditional RON Loss in the Myeloid Compartment Phenocopies Global HGFL Loss in the RON-Driven Mammary Tumorigenesis Model, MMTV-RON

**Figure 2:**
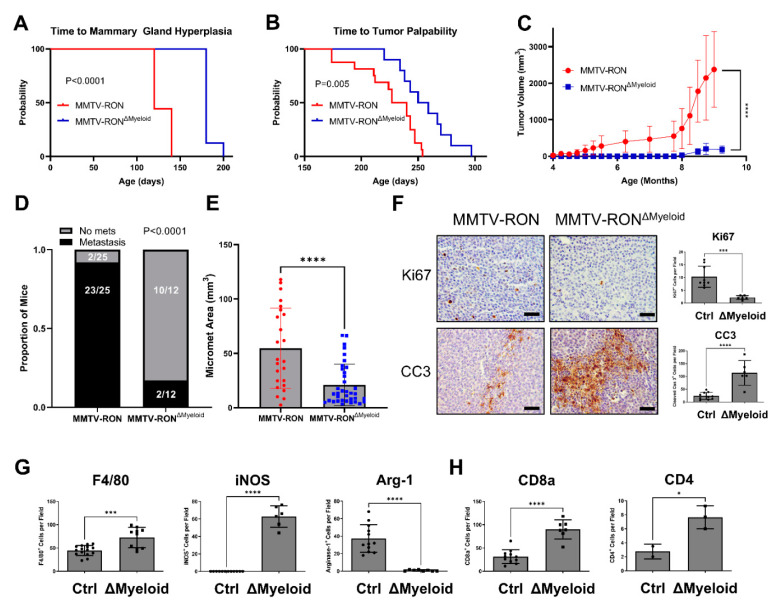
Conditional RON deletion in the myeloid compartment phenocopies global HGFL loss in a RON-driven mammary tumorigenesis model. Kaplan-Meier analysis with Log-Rank statistics of mice for (**A**) time to onset of mammary ductal hyperplasia (MMTV-RON, n = 9; ΔMyeloid, n = 8) and (**B**) time to tumor palpability of MMTV-RON+ mice with or without conditional deletion of RON in the myeloid compartment (ΔMyeloid; MMTV-RON, n = 16; ΔMyeloid, n = 10). (**C**) Tumor growth kinetics of MMTV-RON+ or MMTV-RON ΔMyeloid mice (MMTV-RON, n = 9; ΔMyeloid, n = 9) and (**D**) respective gross metastatic incidence at 90 days of age. (**E**) Micro-metastatic area measured on H&E stained sections from mice of both genotypes taken from mammary tumors with comparable tumor volume (MMTV-RON, n = 6; ΔMyeloid, n = 5). (**F**) Quantitated histological analysis of MMTV-RON and ΔMyeloid mammary tumor sections stained with Ki67, CC3, (**G**) F4/80, iNOS, Arginase-1, (**H**) CD8a, or CD4. Comparisons of histological samples between groups were analyzed using Student’s *t*-tests. * *p* < 0.05; *** *p* < 0.001; **** *p* < 0.0001.

HGFL loss in the PyMT model provided valuable information on the role of coordinate overexpression of RON/HGFL in mammary tumorigenesis [5] but was constrained by the lack of specificity in cell types that may be responding to HGFL either provided directly from the tumor or physiologic sources. Given our previous work demonstrating the consequences of global HGFL loss in the RON-driven MMTV-RON mouse model of breast cancer, we devised a study to examine the macrophage response to HGFL and its contributions to mammary tumorigenesis and metastatic progression. To do this, we crossed MMTV-RON^+^ (transgene), RON floxed (FL/FL; endogenous RON gene) mice with Lysozyme M-Cre (Lys-Cre) mice (a Cre driver that was previously used to delete RON in the myeloid compartment [17,36,37]). These crosses generated mice with a conditional deletion of RON in the myeloid compartment (MMTV-RON^ΔMyeloid^). The resultant mice develop mammary tumors driven by transgenic RON overexpression and display HGFL overexpression [5], but myeloid cells in these animals lack RON expression and do not respond to HGFL. MMTV-RON^ΔMyeloid^ mice have extended time to mammary gland hyperplasia (Figure 2A), extended time to tumor development (Figure 2B), and overall impaired mammary tumor growth kinetics (Figure 2C). The incidence of lung metastases (Figure 2D) and lung metastatic area (Figure 2E) is also reduced in MMTV-RON^ΔMyeloid^ mice when compared with control mice containing a similar tumor burden. MMTV-RON^ΔMyeloid^ tumors have decreased cell proliferation (Ki67^+^ staining) and increased apoptosis (Cleaved Caspase-3^+^ [CC3^+^] staining; Figure 2F). MMTV-RON^ΔMyeloid^ tumors, compared with controls, have increased overall macrophage recruitment based on F4/80^+^ staining, increased M1 macrophage numbers based on iNOS^+^ staining, and decreased M2 macrophages based on Arginase-1^+^ staining (Figure 2G). Further, MMTV-RON^ΔMyeloid^ mammary tumors compared with controls show increased numbers of CD8a^+^ T cells and CD4^+^ T cells (Figure 2H). Taken together, conditional loss of RON in the myeloid compartment results in impaired tumorigenesis, impaired metastatic progression, and enhanced recruitment of immune cell populations similar to mice with a global HGFL deletion in the MMTV-RON model [5].

### 3.3. Tumor Cell-Secreted and Physiologic Sources of HGFL Each Support Mammary Tumor Growth

**Figure 3:**
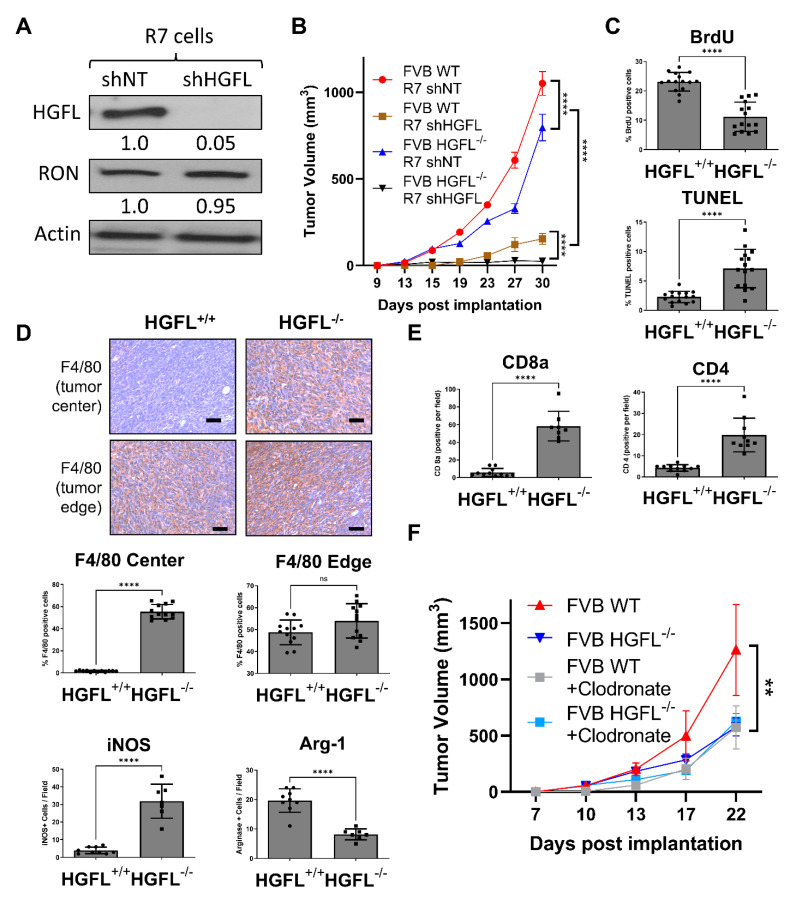
Loss of tumor cell-produced HGFL or systemic (physiologic) HGFL alters mammary tumorigenesis and immune cell recruitment. (**A**) Western blot analysis of lysates from R7 shNT and R7 shHGFL cells for HGFL and Actin (loading control). (**B**) Mammary tumor growth kinetics of R7 shNT and R7 shHGFL cells following orthotopic implantation into syngeneic HGFL deficient (FVB HGFL^−/−^) or HGFL proficient FVB (FVB WT; HGFL^+/+^) mice (n = 5 mice/group) and results were compared with a repeated-measures two-way ANOVA with Holm-Šídák post hoc tests. (**C**) Tumor characterization through histological analyses for Ki67, CC3, (**D**) F4/80, iNOS, Arginase-1, and (**E**) CD8a and CD4. (**F**) Tumor growth kinetics of mammary tumors generated from R7 cells in either WT or HGFL^−/−^ mice with or without liposomal clodronate treatment to deplete macrophages (n = 6 mice/group). Groups were compared using a two-way ANOVA with Holm-Šídák post hoc tests. ** *p* < 0.01; **** *p* < 0.0001; ns, not significant.

The use of conditional RON deletion models has provided valuable insight into cell-type-specific responses to HGFL and ensuing effects on mammary tumor growth. Further, HGFL global deletion demonstrated the pro-tumorigenic role of HGFL-mediated RON activation [5]. However, the pro-tumorigenic potential of individual sources of HGFL (physiologic versus tumor cell-produced) has not been evaluated. To test this, we employed the murine R7 breast cancer cell line which expresses both RON and HGFL [4,7]. The R7 cells were modified with a non-targeting and HGFL targeting shRNA to generate isogenic R7 cell lines with (R7 shNT) and without HGFL (R7 shHGFL) (Figure 3A). Next, we performed syngeneic orthotopic mammary gland transplantation studies whereby R7 control (shNT) and R7 shHGFL cells were placed into the mammary glands of either wild-type (WT) or HGFL deficient (HGFL^−/−^) syngeneic hosts. Control R7 shNT cells efficiently grew mammary tumors in WT mice and showed the greatest extent of mammary tumor growth over time compared to the R7 shHGFL cells transplanted into HGFL^−/−^ hosts which showed virtually no tumor growth (Figure 3B). Moreover, R7 shHGFL cells exhibited limited growth in WT mice and only marginally improved tumor growth over R7 shHGFL cells in HGFL^−/−^ mice. The R7 shHGFL cells grew significantly less compared with R7 shNT cells in HGFL^−/−^ mice. Importantly, these studies show a pivotal functional role for tumor cell-produced HGFL that cannot be compensated by systemic (physiologic) host HGFL expression. These studies suggest that local tumor cell-produced HGFL plays a key role in mammary tumorigenesis (acting on tumor cells and immune cells in the TME) while physiologic sources of HGFL (primarily hepatocyte-derived) are permissive for tumor growth and may sustain the tumor prior to HGFL upregulation in the tumor proper.

As mammary tumor growth for IHC analysis was limited in the R7 shHGFL groups, we examined the contributions of host-produced HGFL to tumor characteristics. Tumor cell proliferation (BrdU^+^ staining) was reduced in R7 shNT tumor-bearing HGFL^−/−^ mice compared with R7 shNT tumor-bearing WT mice complemented by elevated cell death (TUNEL^+^ staining) as depicted in Figure 3C. These data demonstrate that even while tumor cell-produced HGFL is present, loss of physiologic HGFL reduces tumor cell proliferation and enhances cell death. Examination of macrophage recruitment (by F4/80^+^ staining) into the tumor microenvironment showed no differences between groups at the tumor edge (bottom panels, Figure 3D). However, significantly more F4/80^+^ macrophages were found within the mammary tumor centers in R7 shNT cells implanted into HGFL^−/−^ mice compared to cells implanted into HGFL replete mice (Figure 3D). Importantly, M1 iNOS^+^ (M1 macrophage marker) staining was elevated while Arginase-1^+^ staining (M2 macrophage marker) was diminished in the mammary tumors from R7 shNT cells injected into HGFL^−/−^ mice compared to cells transplanted into WT mice (Figure 3D). These data suggest that physiologic HGFL production helps support Arginase-1^+^ macrophages in the TME, and that loss of physiologic produced HGFL leads to a bias towards iNOS^+^ macrophages. Examination of CD4^+^ and CD8a^+^ T cell recruitment showed consistently increased CD4^+^ and CD8a^+^ T cells in tumors with R7 shNT cells transplanted into HGFL^−/−^ mice compared with numbers in WT mice (Figure 3E), suggesting physiologic HGFL suppresses T cell recruitment to tumors.

To determine the functional contribution of macrophages in the context of HGFL, we examined the growth of orthotopically transplanted R7 control cells into WT or HGFL^+/+^ mice and treated the mice with or without clodronate to deplete macrophages (Figure 3F). Mice with HGFL expression (tumor produced and physiologic HGFL) showed the greatest extent of tumor growth whereas HGFL physiologic loss or macrophage depletion showed similarly reduced tumor growth that was indistinguishable from the combined systemic HGFL loss and macrophage depletion. Taken together, these studies suggest that physiologic sources of HGFL support mammary tumorigenesis by promoting tumor cell proliferation, limiting tumor cell death, recruiting M2 (Arginase-1^+^) macrophages, and suppressing CD4^+^ and CD8a^+^ T cell recruitment. These studies also show that macrophages are a key effector of the physiologic sources of HGFL during tumorigenesis as macrophage loss in WT mice phenocopies the growth of mammary tumors in HGFL deficient animals.

### 3.4. Tumor Cell-Secreted HGFL Promotes Macrophage Migration and Mammosphere Formation through Autocrine and Paracrine Mechanisms

**Figure 4:**
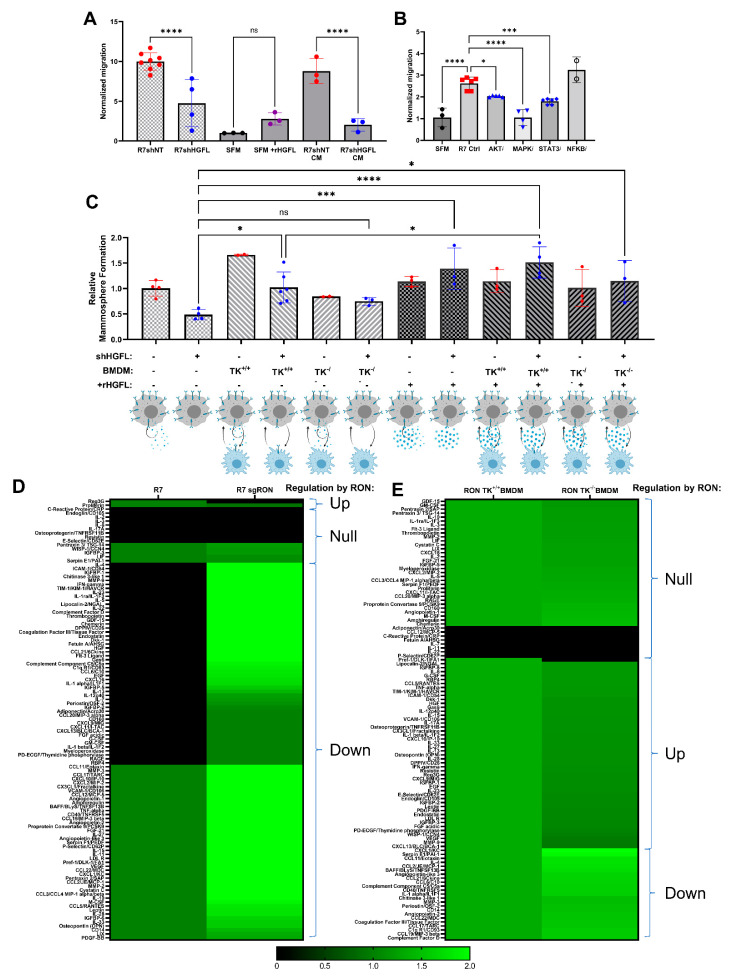
Loss of HGFL in breast cancer cells impairs mammosphere formation, macrophage migration, and alters the secretome of both cell types. (**A**) Macrophage migration towards denoted stimulus including serum-free media (SFM), SFM + recombinant HGFL (rHGFL, 50ng/mL), conditioned media (CM) from R7 control or R7shHGFL cells, or cultured R7 control or R7shHGFL cells. Groups were compared using one-way ANOVA with Holm-Šídák post hoc tests. (**B**) Macrophage migration as in (**A**) toward R7 cells pretreated with vehicle, an Akt inhibitor (AKTi), a MAPK inhibitor (MAPKi), a Stat3 inhibitor (STAT3i), or an NFkB inhibitor (NFKBi). Following inhibitor treatment for 6 h, R7 cells were washed, placed in SFM, and used as a chemoattractant. (**C**) Mammosphere formation of R7 cells with and without HGFL knockdown (shHGFL), monoculture or co-culture with RON TK^+/+^ BMDM, RON TK^−/−^ BMDM, and with and without supplementation with 50 ng/mL recombinant HGFL (rHGFL). Groups were compared using a one-way ANOVA with Holm-Šídák post hoc tests. (**D**,**E**) Heatmaps of secreted proteins detected by Mouse XL Cytokine Arrays incubated with conditioned media from (**D**) R7 cells and R7 sgRON cells (R7 cells with RON loss via CRISPR-Cas9 technology, R7 KD cells from [7]) or (**E**) with conditioned media from WT (RON TK^+/+^) or RON deficient TK^−/−^ BMDM. * *p* < 0.05; *** *p* < 0.001; **** *p* < 0.0001; ns, not significant.

To examine cell-type-specific consequences of tumor cell-secreted HGFL on macrophages, we first evaluated the migration of bone marrow-derived macrophages (BMDMs) toward R7 cells with and without HGFL expression or conditioned media (CM) from R7 cells with and without HGFL expression (Figure 4A). As controls, we used macrophage migration toward serum-free media (SFM) alone or recombinant HGFL (rHGFL) in SFM. As shown in Figure 4A, macrophages migrated significantly toward either R7 control cells or CM from R7 control cells compared to migration toward R7 shHGFL. Interestingly, macrophages did not migrate significantly toward HGFL alone. These data suggest that paracrine HGFL action on macrophage RON is likely not responsible for macrophage recruitment/migration. Rather, autocrine RON activation by tumor cell-produced HGFL leads to the production of tumor cell-secreted factors that then function in paracrine on macrophages to promote macrophage migration. To interrogate intracellular signaling pathway(s) that are important downstream of HGFL-RON signaling in tumor cells that elicit macrophage migration, we repeated the migration assay using R7 cells with and without Akt inhibition (AKTi), ERK1/2 inhibition (MAPKi), Stat3 inhibition (STAT3i) or NFkB inhibition (NFKBi); these pathways are all known targets of HGFL-RON signaling [10]. R7 control cells showed significant induction of macrophage migration over SFM controls. NFKBi showed no significant difference from R7 controls whereas AKTi and STAT3i showed significant reductions; however, MAPKi migration resembled that of SFM (Figure 4B). Taken together, autocrine HGFL-RON signaling in tumor cells leads to the activation of pathways (including MAPK, and partially Akt/Stat3), which support macrophage migration.

We previously showed a role for HGFL-RON signaling in tumor cells and macrophages to promote mammosphere formation/breast cancer stem cells [6,17]. To evaluate the cell type-specific roles of tumor cell-secreted HGFL on mammosphere/breast cancer stem cell formation, we co-cultured HGFL proficient and deficient R7 cells with WT (TK^+/+^) or with RON deficient (TK^−/−^) BMDMs, with or without rHGFL rescue (Figure 4C). HGFL knockdown in R7 cells impaired mammosphere formation compared to R7 controls cells which could be rescued by the addition of HGFL. Addition of TK^+/+^ BMDM but not TK^−/−^ BMDM improved mammosphere formation. Moreover, supplementing with rHGFL improved mammosphere formation of R7 HGFL knockdown cells co-cultured with TK^+/+^ BMDM. These data support a model wherein tumor cell-secreted HGFL activates RON in both tumor cells (autocrine activation) and macrophages (paracrine activation), which each promote mammosphere formation.

### 3.5. RON Signaling Induces Changes to the Secretome of Tumor Cells and Macrophages

Functional analyses suggest changes in secreted factors from both tumor cells (to alter macrophage migration) and from macrophages (to alter mammosphere formation). Next, we utilized an unbiased approach to characterize secretome changes in both cell types using CM from R7 cells or R7 RON-targeted CRISPR clone, R7 sgRON (previously characterized [7]; Figure 4D) or CM from TK^+/+^ BMDM or TK^−/−^ BMDM (results are summarized in heatmap form in Figure 4D,E). Numerous changes based on HGFL-RON signaling are downregulated (e.g., lipocalin-2 [LCN2]) and a few upregulated (regenerating family member 3 gammas [Reg3G]) in tumor cells. In macrophages, numerous chemokines (CXCL family) and cytokines (interleukin family) are upregulated by HGFL-RON signaling, with some downregulated (complement proteins). Taken together, HGFL-RON signaling in both tumor cells and macrophages alters the secretome in a manner to support tumor growth and progression.

### 3.6. Inhibiting HGFL-RON Signaling in Tumor Cells and Macrophages via BMS777607 Impairs Tumor Growth, Metastasis, and Tumoral Macrophage Recruitment

**Figure 5:**
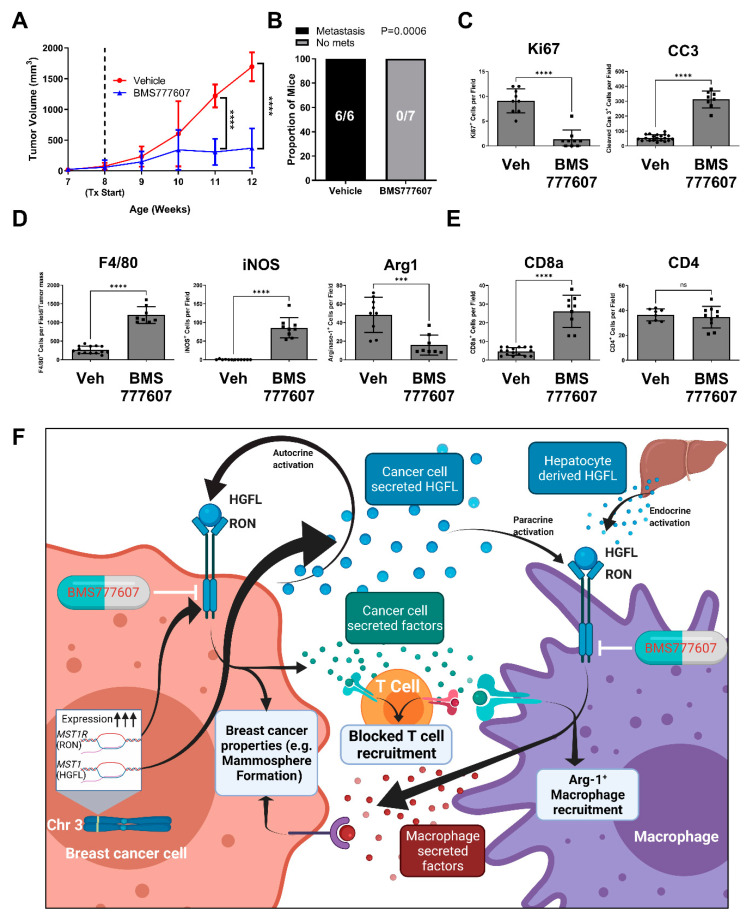
Pharmacological inhibition of HGFL-RON signaling via BMS777607 treatment abrogates PyMT-driven tumorigenesis and metastasis. (**A**) Tumor growth kinetics of mammary tumors in PyMT+ mice treated with 50 mg/kg/day BMS777607 versus vehicle control (Vehicle: n = 6; BMS777607: n = 7). Data was analyzed with a two-way ANOVA with Holm-Šídák post hoc tests. (**B**) Metastatic incidence in PyMT+ mice from (**A**) with BMS777607 treatment versus vehicle control. Data was analyzed using Fisher’s exact test. (**C**) Histological analysis of BMS777607 treated and vehicle-treated PyMT tumor sections stained with Ki67, CC3, (**D**) F4/80, iNOS, Arginase 1, (**E**) CD8a, or CD4. (**F**) Working model of our experimental findings summarized (generated using BioRender). *** *p* < 0.001; **** *p* < 0.0001; ns, not significant.

Our data demonstrates a strong role for HGFL-RON signaling in both tumor cells and macrophages to support breast cancer growth and progression. To examine the pharmacological effects of HGFL-RON signaling inhibition in vivo, we utilized the PyMT model and the RON inhibitor, BMS777607. Tumor growth kinetics showed a significant reduction and plateau of tumor growth in BMS777607 treated mice relative to vehicle-treated controls (Figure 5A). BMS777607 treated mice showed no discernable metastasis, whereas all vehicle-treated mice had metastasis (Figure 5B). Furthermore, tumors from BMS777607 treated mice show a significant reduction in cell proliferation (Ki67^+^) and induction of apoptosis (CC3^+^) relative to tumors from vehicle-treated mice (Figure 5C). HGFL-RON inhibition showed a consistent macrophage recruitment pattern to our previous models wherein total macrophage numbers (F4/80^+^) and iNOS^+^ staining (M1 macrophages) is increased in tumors from BMS777607 treated mice while Arginase-1^+^ staining M2 macrophages are reduced relative to tumors from the vehicle-treated mice (Figure 5D). T cell recruitment patterns also match our previous models wherein CD8a^+^ T cell recruitment is enhanced in tumors from BMS777607 treated mice compared with tumors from vehicle-treated mice (Figure 5E). Taken together, the use of BMS777607 to inhibit HGFL-RON signaling shows consistent results with our genetic targeting approaches in clinically relevant models, thus providing preclinical evidence of BMS777607 as a RON-targeted therapeutic. Figure 5F contains a model summarizing our findings.

### 3.7. MST1R (RON) and MST1 (HGFL) Show Correlated Gene Expression in Virtually All Tumor Types including Breast Cancer

**Figure 6:**
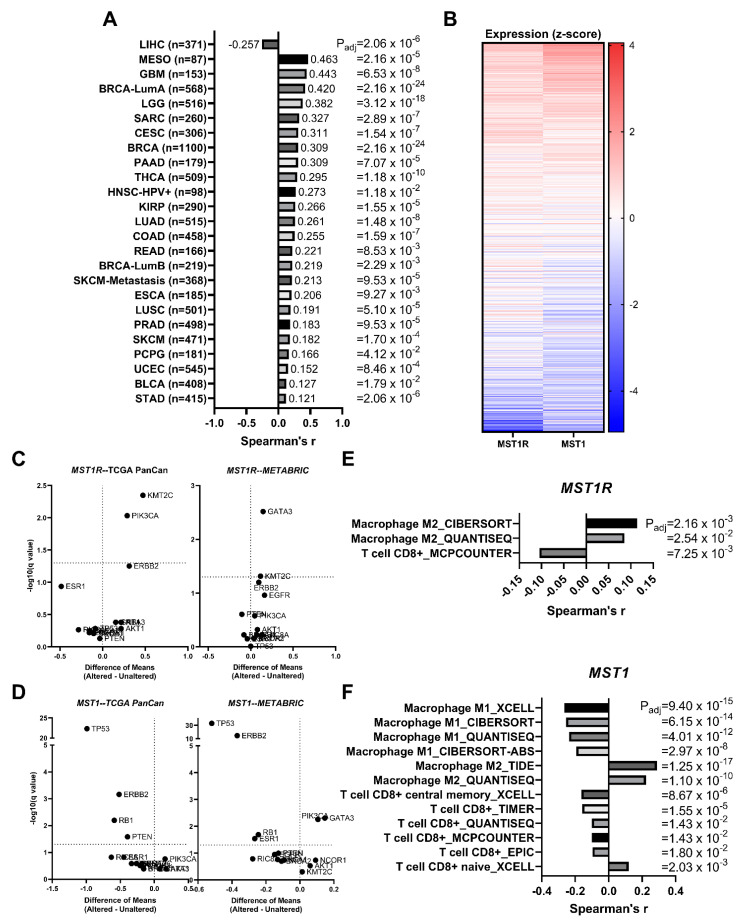
RON (*MST1R*) and HGFL (*MST1*) expression are correlated in virtually all tumor types and expression is not associated with recurrent breast cancer driver mutations. (**A**) *MST1R* and *MST1* gene expression are coordinately upregulated across specified cancer types from The Cancer Genome Atlas (TCGA) evaluated with Spearman’s correlation. (**B**) *MST1R* and *MST1* RNA expression from TCGA showing coordinate expression in heatmap form. Volcano plots of (**C**) *MST1R* and (**D**) *MST1* RNA expression stratified between Altered and Unaltered samples of listed recurrent breast cancer driver mutations from TCGA Pan-Cancer and METABRIC datasets were evaluated using Multiple *t*-tests on log-transformed expression values. Significant correlations of specified immune cell infiltration signatures with (**E**) *MST1R* and (**F**) *MST1* gene expression using the tumor immune microenvironment estimation resource (TIMER) in breast cancer data from TCGA.

We analyzed data from The Cancer Genome Atlas (TCGA) Pan-Cancer (PanCan) datasets and found a significant positive correlation between *MST1R* and *MST1* expression in all available cancer type datasets except liver hepatocellular carcinoma (LIHC), where *MST1R/MST1* are negatively correlated (Figure 6A). These data include breast cancer samples whereby the coordinate expression of *MST1R* and *HGFL* is represented in the heatmap in Figure 6B.

### 3.8. MST1R and MST1 Genes Are Virtually Never Altered/Mutated in Breast Cancer, and MST1R/MST1 Expression Is Not Associated with Any Recurrent Breast Cancer Driver Alterations/Mutations

Neither *MST1R* nor *MST1* mutational drivers, amplification events, or other genomic alterations have been extensively reported. To ascertain the frequency of such alterations, we gathered data from numerous publicly available datasets which showed coordinate alterations of *MST1R*/*MST1* in 0.7% of breast tumors analyzed, 0.5% with *MST1R* alone, 0.3% with *MST1* alone, and 98.5% with neither altered; Chi-square analysis supports that *MST1R*/*MST1* alterations tend to co-occur in the rare event that they do occur (Table 1). We next asked whether genomic alterations known to drive breast cancer have associations with increased *MST1R*/*MST1* expression. A recent publication identified driver gene mutations/alterations associated with metastatic breast cancer [38]. We used nine driver genes identified in this study (*TP53*, *ESR1*, *GATA3*, *KMT2C*, *NCOR1*, *AKT1*, *NF1*, *RIC8A*, *RB1*) as well as additional genes frequently altered in breast cancer (*PTEN*, *PIK3CA*, *BRCA1*, *BRCA2*, *ERBB2*, *EGFR*). Driver gene alteration status (Altered or Unaltered) was used to stratify tumor samples, and the difference in means of *MST1R* (Figure 6C) and *MST1* (Figure 6D) expression individually were compared across Altered/Unaltered status using samples from the TCGA PanCan and METABRIC datasets. There were no hits consistent across both genes and both datasets. However, *GATA3* alterations were identified between *MST1R* and *MST1* in the METABRIC dataset. *KMT2C* was a consistent hit between both datasets for *MST1R* but not *MST1*, and *PIK3CA* was an *MST1R* hit in TCGA PanCan and an *MST1* hit in the METABRIC data. Interestingly, *TP53*, *ERBB2*, and *RB1* were consistent hits where the Unaltered (or wild-type) gene shows higher *MST1* expression, implying that alteration in these genes is associated with reduced *MST1* expression relative to the wild-type. Taken together, no hits were consistent across both *MST1R* and *MST1* within a single dataset.

### 3.9. MST1R and MST1 Genes Are Correlated with M2 Macrophage Infiltration Gene Signatures and Anti-Correlated with CD8^+^ T Cell Infiltration Gene Signatures in Human Breast Cancer Patient Samples

A significant positive correlation of *MST1R* and *MST1* with M2 macrophage infiltration and naïve T cell responses was identified by examining the tumor immune microenvironment estimation resource (TIMER) from breast cancer data from TCGA with *MST1R* (Figure 6E) and *MST1* (Figure 6F) expression. A significant negative correlation of *MST1* with M1 macrophage infiltration and activated T cell responses were also observed. This correlation of *MST1R* and *MST1* with M2 polarized macrophages is further supported by data in Figure 1F.

## 4. Discussion

Our preclinical experiments began with the observation that RON and HGFL become overexpressed in the PyMT murine autochthonous tumor model, thus making it a good model to study the contribution of HGFL to tumorigenesis. The resulting cross of HGFL^−/−^ mice into the PyMT model showed a significant impairment of tumor growth, metastasis, and tumor characteristics that were repeatedly observed in several in vivo experiments upon loss of HGFL including reduced tumor cell proliferation, increased tumor cell death, elevated M1 (iNOS^+^) macrophage recruitment, diminished M2 (Arginase-1^+^) macrophage recruitment, and increased CD8a^+^ and CD4^+^ T cell recruitment. Our previous study using the RON-driven MMTV-RON breast tumor model crossed with HGFL^−/−^ mice yielded nearly identical results to the myeloid RON deletion in MMTV-RON^+^ mice employed herein [5]. Similar studies evaluating myeloid and macrophage-specific RON deletion in the PyMT model strongly suggest that macrophages are the most logical paracrine effector cells to tumor cell-secreted HGFL. While important, models with global HGFL loss did not provide insight into the functional contribution of different HGFL sources.

The use of HGFL-modulated tumor cells in HGFL-modulated mice revealed the phenotypic contributions of the different sources (tumor cell-secreted and physiologic) of HGFL, where complete ablation of HGFL has the strongest anti-tumor response, followed by loss of HGFL in tumor cells, loss of physiologic HGFL, and robust tumor growth when both HGFL sources are intact. While tumor growth of HGFL-proficient tumors in HGFL-deficient mice was only modestly reduced, tumor characteristics including alterations to total/M1/M2 macrophage recruitment resembled our genetically engineered mouse models. This suggests that physiologic sources of HGFL play a role in altering the macrophage composition in the tumor microenvironment (TME). To further test the functional requirement of these macrophages in the TME, macrophage depletion (via clodronate) was overlayed upon the orthotopic model testing physiologic HGFL, and the results of this experiment suggest physiologic HGFL and ensuing alterations to macrophage composition in the TME indeed supports tumor growth.

Given the large effect that loss of tumor cell-secreted HGFL has on tumor growth, we used in vitro systems to examine the interaction of tumor cells producing HGFL, ensuing effects on macrophages, and how these macrophages support the tumor cell. The data herein, and supported by prior studies [5,7,17], demonstrate that HGFL itself does not promote macrophage migration but that HGFL-dependent alterations to the tumor cell secretome promote macrophage migration, and that these alterations are MAPK-dependent, with possible Akt/Stat3 roles. These data are supported by a screen of inhibitors targeting known pathways activated by RON. Mammosphere formation assays including both monoculture and co-culture with RON proficient or RON deficient bone marrow-derived macrophages (BMDM) recapitulated several points, including that loss of HGFL in tumor cells impairs mammosphere formation [6], and that addition of RON proficient, but not RON deficient BMDM augments mammosphere formation [17]. Additionally, we showed that HGFL deficient tumor cell phenotypes can be rescued by exogenous HGFL supplementation, and that augmented mammosphere formation when co-cultured with RON proficient BMDM requires HGFL stimulation. Given that the various in vivo models demonstrate a role for RON in the myeloid compartment (largely attributable to macrophages), the in vitro data support this, as RON/HGFL overexpressing tumor cells co-cultured with RON deficient macrophages do not show a boost to mammosphere formation even when exogenous HGFL is provided. Conversely, RON proficient macrophages promote mammosphere formation, and when HGFL deficient tumor cells are provided exogenous HGFL in co-culture with macrophages, enhanced mammosphere formation is also seen. Thus, tumor cell produced HGFL acts in an autocrine fashion to promote macrophage recruitment to the tumor via MAPK-dependent alterations to the secretome (and autonomously promote mammosphere formation), and in a paracrine fashion to elicit HGFL-RON-dependent alterations to the macrophage secretome that promote mammosphere formation of RON/HGFL expressing tumor cells.

These preclinical models have allowed for deeper dissection of cell-type-specific contributions of HGFL-RON signaling from physiologic and tumor cell sources, with a demonstrable pro-tumor function for each component. Finally, we tested the use of the HGFL-RON signaling inhibitor, BMS777607 in the PyMT model. BMS777607 has completed Phase I testing showing safe dosing and oral bioavailability [39]. BMS777607 treatment led to a plateau of tumor volume two weeks after the first dose was administered and abrogated metastasis in treated mice. Alterations of tumor characteristics matched our other models except for a lack of change in CD4^+^ T cell recruitment. Nonetheless, our data suggest the use of BMS777607 strongly affects tumorigenesis and metastasis in a model exhibiting RON and HGFL overexpression.

Studies herein are the first to report correlated co-expression of *MST1R* and *MST1* across nearly all cancer types and are consistent with a previous study that reported a lack of alterations using polymerase chain reaction-single strand conformational polymorphism (PCR-SSCP) analysis [40]. *MST1R*/*MST1* alterations were observed in an extreme minority of samples and Chi-square analysis supported that those alterations in *MST1R/MST1* typically co-occur. Moreover, out of a panel of known mutational drivers of breast cancer, there were no hits consistent in both TCGA PanCan and METABRIC datasets for both genes. *KMT2C* was a consistent hit across TCGA PanCan and METABRIC datasets but for *MST1R* only, thus potentially representing a mechanism by which *MST1R* alone becomes overexpressed in breast cancer. There were no hits in this manner for *MST1* alone.

## 5. Conclusions

We conclude that RON and HGFL co-overexpression within tumor cells drives aggressive breast tumorigenesis through autocrine and paracrine mechanisms affecting both the tumor cells and tumor-associated macrophages and HGFL-RON signaling activation alters the secretome in both tumor cells and macrophages. In addition to tumor cell-produced HGFL, physiologic sources of HGFL support aggressive breast tumorigenesis. Finally, RON inhibition using the pharmacologic inhibitor, BMS777607, abrogates tumor growth and progression of RON/HGFL overexpressing tumors.

## Figures and Tables

**Table 1:** Genetic/genomic alterations affecting *MST1R* and *MST1* are rare but co-occur. Specified datasets available on cBioPortal were queried for *MST1R* and *MST1* mutations/alterations.

Queried Datasets	Number of Samples with Alterations in Specified Gene(s)				Statistics			
	*MST1R*	*MST1*	Both	Neither	*p*-Value	Q-Value	Log_2_ Odds Ratio	Tendency
TCGA PanCan, MBC Provisional, MBC INSERM	7	5	10	1483	<0.001	<0.001	>3	Co-occurrence

## Data Availability

METABRIC and TCGA data are available through cBioPortal [34].

## Supplementary Materials

The following are available online at https://www.mdpi.com/article/10.3390/cancers14102493/s1, Figure S1: Full Western blot images.

## References

[1] Mallakin A., Kutcher L.W., McDowell S.A., Kong S., Schuster R., Lentsch A.B., Aronow B.J., Leikauf G.D., Waltz S.E. (2006). Gene expression profiles of Mst1r-deficient mice during nickel-induced acute lung injury. Am. J. Respir. Cell Mol. Biol..

[2] Stuart W.D., Kulkarni R.M., Gray J.K., Vasiliauskas J., Leonis M.A., Waltz S.E. (2011). Ron receptor regulates Kupffer cell-dependent cytokine production and hepatocyte survival following endotoxin exposure in mice. Hepatology.

[3] Li J., Chanda D., Shiri-Sverdlov R., Neumann D. (2015). MSP: An emerging player in metabolic syndrome. Cytokine Growth Factor Rev..

[4] Zinser G.M., Leonis M.A., Toney K., Pathrose P., Thobe M., Kader S.A., Peace B.E., Beauman S.R., Collins M.H., Waltz S.E. (2006). Mammary-specific Ron receptor overexpression induces highly metastatic mammary tumors associated with beta-catenin activation. Cancer Res..

[5] Benight N.M., Wagh P.K., Zinser G.M., Peace B.E., Stuart W.D., Vasiliauskas J., Pathrose P., Starnes S.L., Waltz S.E. (2015). HGFL supports mammary tumorigenesis by enhancing tumor cell intrinsic survival and influencing macrophage and T-cell responses. Oncotarget.

[6] Ruiz-Torres S.J., Benight N.M., Karns R.A., Lower E.E., Guan J.L., Waltz S.E. (2017). HGFL-mediated RON signaling supports breast cancer stem cell phenotypes via activation of non-canonical beta-catenin signaling. Oncotarget.

[7] Bourn J.R., Ruiz-Torres S.J., Hunt B.G., Benight N.M., Waltz S.E. (2021). Tumor cell intrinsic RON signaling suppresses innate immune responses in breast cancer through inhibition of IRAK4 signaling. Cancer Lett..

[8] Eyob H., Ekiz H.A., DeRose Y.S., Waltz S.E., Williams M.A., Welm A.L. (2013). Inhibition of Ron kinase blocks conversion of micrometastases to overt metastases by boosting anti-tumor immunity. Cancer Discov..

[9] Babicky M.L., Harper M.M., Chakedis J., Cazes A., Mose E.S., Jaquish D.V., French R.P., Childers B., Alakus H., Schmid M.C. (2019). MST1R kinase accelerates pancreatic cancer progression via effects on both epithelial cells and macrophages. Oncogene.

[10] Wagh P.K., Peace B.E., Waltz S.E. (2008). Met-related receptor tyrosine kinase Ron in tumor growth and metastasis. Adv. Cancer Res..

[11] Gurusamy D., Ruiz-Torres S.J., Johnson A.L., Smith D.A., Waltz S.E. (2014). Hepatocyte growth factor-like protein is a positive regulator of early mammary gland ductal morphogenesis. Mech. Dev..

[12] Yao H.P., Zhou Y.Q., Zhang R., Wang M.H. (2013). MSP-RON signalling in cancer: Pathogenesis and therapeutic potential. Nat. Rev. Cancer.

[13] Maggiora P., Marchio S., Stella M.C., Giai M., Belfiore A., De Bortoli M., Di Renzo M.F., Costantino A., Sismondi P., Comoglio P.M. (1998). Overexpression of the RON gene in human breast carcinoma. Oncogene.

[14] Hunt B.G., Wicker C.A., Bourn J.R., Lower E.E., Takiar V., Waltz S.E. (2020). MST1R (RON) expression is a novel prognostic biomarker for metastatic progression in breast cancer patients. Breast Cancer Res. Treat..

[15] Lee W.Y., Chen H.H., Chow N.H., Su W.C., Lin P.W., Guo H.R. (2005). Prognostic significance of co-expression of RON and MET receptors in node-negative breast cancer patients. Clin. Cancer Res..

[16] Peace B.E., Toney-Earley K., Collins M.H., Waltz S.E. (2005). Ron receptor signaling augments mammary tumor formation and metastasis in a murine model of breast cancer. Cancer Res..

[17] Ruiz-Torres S.J., Bourn J.R., Benight N.M., Hunt B.G., Lester C., Waltz S.E. (2022). Macrophage-mediated RON signaling supports breast cancer growth and progression through modulation of IL-35. Oncogene.

[18] Bezerra J.A., Witte D.P., Aronow B.J., Degen S.J. (1993). Hepatocyte-specific expression of the mouse hepatocyte growth factor-like protein. Hepatology.

[19] Vasiliauskas J., Nashu M.A., Pathrose P., Starnes S.L., Waltz S.E. (2014). Hepatocyte growth factor-like protein is required for prostate tumor growth in the TRAMP mouse model. Oncotarget.

[20] Fluck M.M., Schaffhausen B.S. (2009). Lessons in signaling and tumorigenesis from polyomavirus middle T antigen. Microbiol. Mol. Biol. Rev..

[21] Regua A.T., Arrigo A., Doheny D., Wong G.L., Lo H.W. (2021). Transgenic mouse models of breast cancer. Cancer Lett..

[22] Mantovani A., Sozzani S., Locati M., Allavena P., Sica A. (2002). Macrophage polarization: Tumor-associated macrophages as a paradigm for polarized M2 mononuclear phagocytes. Trends Immunol..

[23] Pan Y., Yu Y., Wang X., Zhang T. (2020). Tumor-Associated Macrophages in Tumor Immunity. Front. Immunol..

[24] Gurusamy D., Gray J.K., Pathrose P., Kulkarni R.M., Finkleman F.D., Waltz S.E. (2013). Myeloid-specific expression of Ron receptor kinase promotes prostate tumor growth. Cancer Res..

[25] Brown N.E., Paluch A.M., Nashu M.A., Komurov K., Waltz S.E. (2018). Tumor Cell Autonomous RON Receptor Expression Promotes Prostate Cancer Growth Under Conditions of Androgen Deprivation. Neoplasia.

[26] Kocher T., Asslaber D., Zaborsky N., Flenady S., Denk U., Reinthaler P., Ablinger M., Geisberger R., Bauer J.W., Seiffert M. (2016). CD4+ T cells, but not non-classical monocytes, are dispensable for the development of chronic lymphocytic leukemia in the TCL1-tg murine model. Leukemia.

[27] Faustino-Rocha A., Oliveira P.A., Pinho-Oliveira J., Teixeira-Guedes C., Soares-Maia R., da Costa R.G., Colaco B., Pires M.J., Colaco J., Ferreira R. (2013). Estimation of rat mammary tumor volume using caliper and ultrasonography measurements. Lab. Anim..

[28] Schneider C.A., Rasband W.S., Eliceiri K.W. (2012). NIH Image to ImageJ: 25 years of image analysis. Nat. Methods.

[29] Kulkarni R.M., Stuart W.D., Gurusamy D., Waltz S.E. (2014). Ron receptor signaling is protective against DSS-induced colitis in mice. Am. J. Physiol. Gastrointest. Liver Physiol..

[30] Ito Y., Teitelbaum S.L., Zou W., Zheng Y., Johnson J.F., Chappel J., Ross F.P., Zhao H. (2010). Cdc42 regulates bone modeling and remodeling in mice by modulating RANKL/M-CSF signaling and osteoclast polarization. J. Clin. Investig..

[31] Liu J., Lichtenberg T., Hoadley K.A., Poisson L.M., Lazar A.J., Cherniack A.D., Kovatich A.J., Benz C.C., Levine D.A., Lee A.V. (2018). An Integrated TCGA Pan-Cancer Clinical Data Resource to Drive High-Quality Survival Outcome Analytics. Cell.

[32] Curtis C., Shah S.P., Chin S.F., Turashvili G., Rueda O.M., Dunning M.J., Speed D., Lynch A.G., Samarajiwa S., Yuan Y. (2012). The genomic and transcriptomic architecture of 2,000 breast tumours reveals novel subgroups. Nature.

[33] Cerami E., Gao J., Dogrusoz U., Gross B.E., Sumer S.O., Aksoy B.A., Jacobsen A., Byrne C.J., Heuer M.L., Larsson E. (2012). The cBio cancer genomics portal: An open platform for exploring multidimensional cancer genomics data. Cancer Discov..

[34] cBioPortal. https://www.cbioportal.org/.

[35] Li T., Fu J., Zeng Z., Cohen D., Li J., Chen Q., Li B., Liu X.S. (2020). TIMER2.0 for analysis of tumor-infiltrating immune cells. Nucleic Acids Res..

[36] Shi J., Hua L., Harmer D., Li P., Ren G. (2018). Cre Driver Mice Targeting Macrophages.

[37] Nikolaidis N.M., Kulkarni R.M., Gray J.K., Collins M.H., Waltz S.E. (2011). Ron receptor deficient alveolar myeloid cells exacerbate LPS-induced acute lung injury in the murine lung. Innate Immun..

[38] Bertucci F., Ng C.K.Y., Patsouris A., Droin N., Piscuoglio S., Carbuccia N., Soria J.C., Dien A.T., Adnani Y., Kamal M. (2019). Genomic characterization of metastatic breast cancers. Nature.

[39] Andrade K., Fornetti J., Zhao L., Miller S.C., Randall R.L., Anderson N., Waltz S.E., McHale M., Welm A.L. (2017). RON kinase: A target for treatment of cancer-induced bone destruction and osteoporosis. Sci. Transl. Med..

[40] Ren X., Daa T., Yada N., Kashima K., Fujitomi Y., Yokoyama S. (2012). Expression and mutational status of RON in neoplastic lesions of the breast: Analysis of MSP/RON signaling in ductal carcinoma in situ and invasive ductal carcinoma. APMIS.

