# Supplementary material for: RON (MST1R) and HGFL (MST1) Co-Overexpression Supports Breast Tumorigenesis through Autocrine and Paracrine Cellular Crosstalk
Source: Cancers (Basel). 2022 May 19;14(10):2493. doi: 10.3390/cancers14102493 (PMC9140067; doi:10.3390/cancers14102493)
Supplement: Supplementary file 1 [file cancers-14-02493-s001.zip › cancers-1692071-supplementary.pdf]

cancers

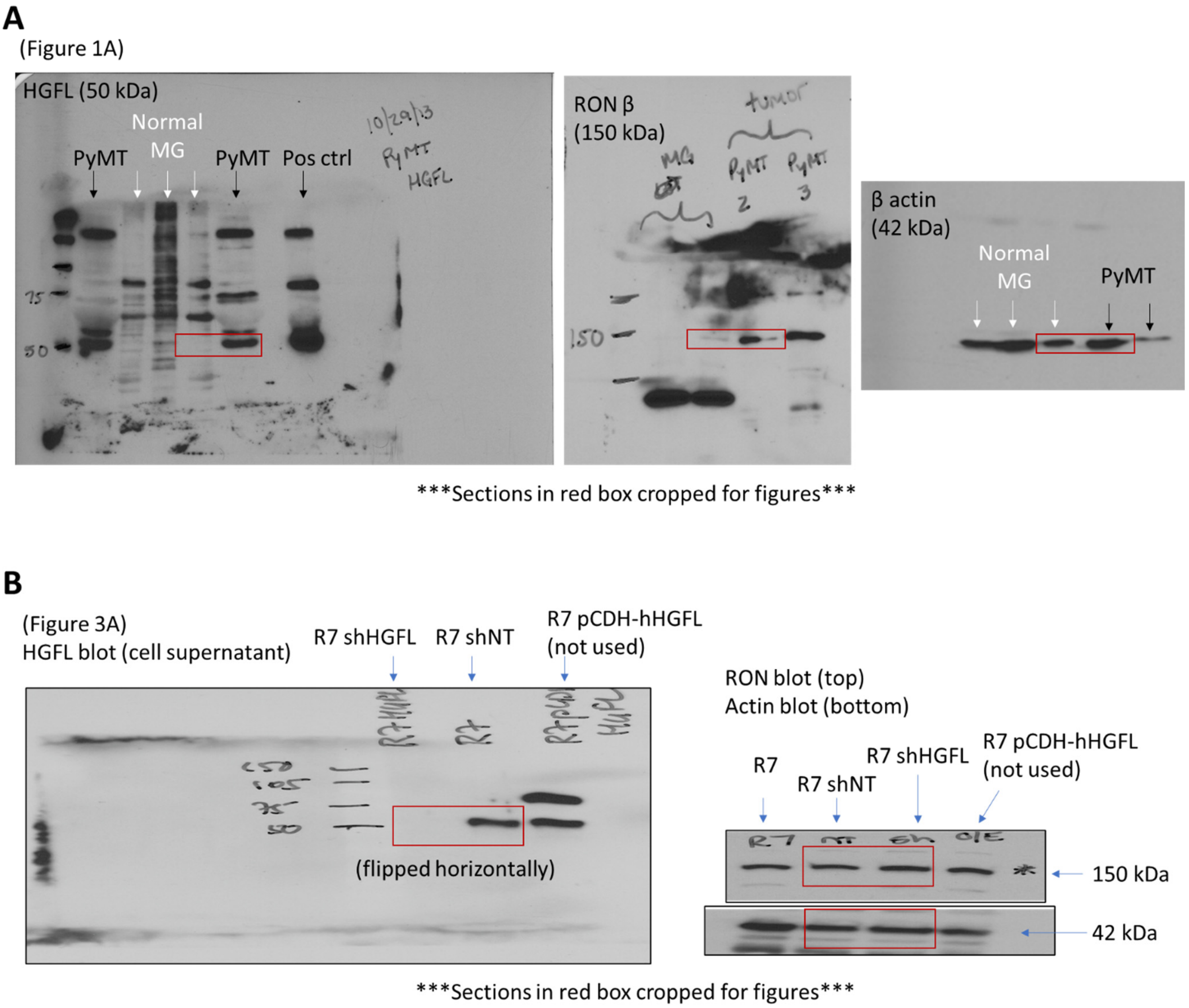

Figure S1. Full western blot images. Images appearing in (A) Figure 1A and (B) Figure 3A.
